# Predictors of response to cardiac resynchronization therapy in patients with chronic right ventricular pacing

**DOI:** 10.1007/s00392-020-01785-9

**Published:** 2020-12-15

**Authors:** Benjamin Rath, Kevin Willy, Julian Wolfes, Christian Ellermann, Florian Reinke, Julia Köbe, Lars Eckardt, Gerrit Frommeyer

**Affiliations:** Department of Cardiology II (Electrophysiology), University Hospital, Albert-Schweitzer-Campus 1, 48149 Münster, Germany

**Keywords:** CRT, Chronic right ventricular pacing, NICM

## Abstract

**Background:**

The benefits of de novo cardiac resynchronization therapy (CRT) in patients with QRS-prolongation and impaired left-ventricular function (LVEF) are well established. Current guidelines also recommend CRT-upgrade in patients requiring permanent or frequent right ventricular pacing (RVP) with symptomatic heart failure and reduced LVEF. Whereas several predictors of response to de novo CRT-implantation such as female gender, QRS-duration, non-ischemic cardiomyopathy (NICM) are known due to large prospective trials, similar factors regarding CRT-upgrade are currently lacking.

**Methods and results:**

We examine 114 patients 3–6 months after CRT-upgrade due to frequent RVP (> 50%) and symptomatic heart failure. Response to CRT was evaluated by improvement in NYHA class referring to the Minnesota Living With Heart Failure Questionnaire. Only cardiomyopathy type and use of Angiotensin-converting-enzyme (ACE) inhibitor had an impact on response to CRT-upgrade in a linear regression model. Patients with NICM presented a greater responder rate than patients with ischemic cardiomyopathy (ICM) (80.4 vs. 60.3%, *p* < 0.05). Other traditional response predictors in de novo CRT recipients (e.g. QRS-width, female gender) showed no effect on CRT-response in this cohort.

**Conclusion:**

Only underlying heart disease (NICM vs. ICM) and the use of ACE inhibitor were significant predictors of response to CRT-upgrade. In contrast to de novo CRT-recipients, where pre-implant QRS-duration is a key predictor, QRS-duration during RV-pacing has no significant impact on CRT-response in this cohort.

**Graphic abstract:**

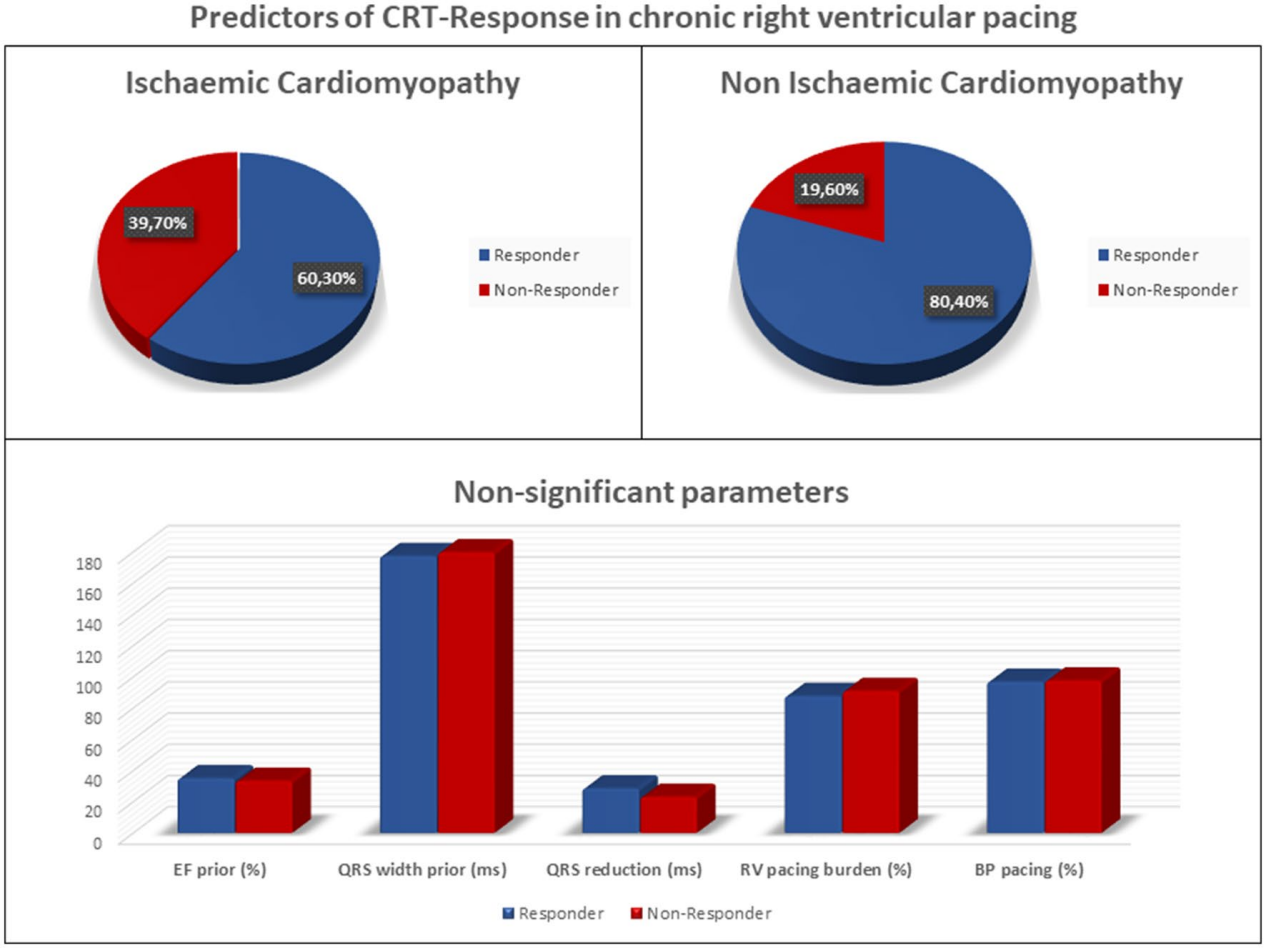

## Introduction

Cardiac resynchronization therapy (CRT) is an established treatment for selected patients with heart failure (HF) and prolonged QRS-duration [1]. The vast majority of patients enrolled in clinical CRT trials had de novo implants and benefits of CRT are especially established in patients with native left bundle branch block (LBBB) [2–4]. International guidelines also recommend CRT-upgrade in patients requiring permanent or frequent RV pacing (RVP) who have symptomatic HF and reduced left ventricular ejection fraction (LVEF) [1]. Current non-randomized studies showed conflicting results in terms of the outcome of CRT-upgrade compared with de novo implantation [5–7].

Furthermore, in contrast to patients with intrinsic LBBB where several predictors of response to CRT (female gender, QRS-duration, non-ischemic cardiomyopathy) are available due to large prospective trials [8], similar predictors regarding CRT-upgrade are currently lacking.

In the current study, we sought to determine predictors of response to CRT-upgrade in patients with chronic RV-pacing.

## Methods

A consecutive cohort of patients who underwent CRT-upgrade due to chronic right ventricular pacing and reduced LVEF (< 50%) between 2013 and 2019 was analyzed. Clinical, ECG and echocardiographic data were evaluated. Chronic RVP was defined as a RV pacing burden of at least 50% pacing on the pre-CRT upgrade device check. All CRT device implantations were performed via a transvenous access targeting a lateral or posterolateral vein for the left ventricular lead position. RV leads were positioned in the RV apex or septum. Various transvenous delivery systems, LV and RV leads, and generators left to the operator’s discretion were used. Whenever an indication for a defibrillator was present, patients received a combined device. For inclusion in the final cohort, only patients with a biventricular pacing percentage of at least 95% were considered. Optimization of the programmed atrioventricular and interventricular delay of the CRT device was performed according to standard protocols of our center.

Patients were reassessed, including device interrogation, in our outpatient clinic 1, 3 and 6 months after CRT implantation.

Response to CRT was defined as improvement in New York Heart Association (NYHA) functional class (≥ 1/2 class) within 3-month follow-up referring to the Minnesota Living With Heart Failure questionnaire [9]. In addition, cardiac decompensations and hospitalizations due to heart failure were quoted. Patients without improvement or even worsening of NYHA class, or at least 1 cardiac decompensation with hospitalization, were classified as nonresponders.

SPSS software (version 26.0; SPSS Inc., Chicago, IL, USA) was used for statistical analysis and database management. For comparison of means, the student *t* test was used for paired or unpaired observations, as appropriate. The χ^2^ test was applied for comparison of proportions between groups. To determine independent predictors of clinical response to CRT, binary logistic regression models were used while CRT response (yes; no) was set as the dependent variable. A *p *value < 0.05 was considered statistically significant.

## Results

### Patients’ characteristics

Our study population consisted of 88 men (77.2%) and 26 women (mean age 72.5 years). Mean NYHA class was 2.93 prior to implantation, with the majority of patients (78.1%) in NYHA class III. The underlying etiology of heart failure was coronary artery disease (CAD) in 63 patients (55.3%) and non-ischemic cardiomyopathy (NICM) in 51 patients (44.7%). Mean EF was 34.5% prior to CRT implantation and mean RV-paced burden was 88.6%. 80 patients (70.18%) received a combined CRT-defibrillator-system. Data regarding the duration of RV-pacing before CRT-upgrade were available in 57 patients (49.56%) with an average duration of 31.92 months. The baseline characteristics are listed in Table 1.

**Table 1:** Baseline characteristics

	Total (*n* = 114)
Gender	
Male	88 (77.19%)
Female	26 (22.81%)
Age (years)	72.48
NYHA class prior to CRT	2.93
II	7 (6.14%)
II–III	10 (8.77%)
III	89 (78.07%)
III–IV	8 (7.02%)
Heart failure etiology	
Non Ischaemic cardiomyopathy	51 (44.74%)
Ischaemic cardiomyopathy	63 (55.26%)
CRT-device	
Defibrillator	80 (70.18%)
Pacemaker	34 (29.82%)
Ejection fraction (%)	34.51
QRS duration prior to CRT (ms)	178.07
QRS duration post CRT (ms)	151.23
Right ventricular pacing burden (%)	88.58
Laboratory results	
N + (mmol/l)	140.13
K + (mmol/l)	4.46
Creatinine (mg/dl)	1.51
Medication	
ß-blockers	105 (92.11%)
ACE-inhibitor	99 (86.84%)
MRA-antagonist	58 (50.88%)

### Response to CRT

In total, 79 patients (69.3%) fulfilled our predefined criterion for a response to CRT (responders), and 35 patients (30.7%) were classified as nonresponders. All patients were alive at 3- and 6-month follow-ups. Responders showed a mean improvement in NYHA class from 2.90 to 2.16. Transthoracic echocardiography during follow-up was performed in 39 patients (33.91%). If echocardiographic data were available, there was a significant correlation between the clinical definition of response and improvement of LVEF (+ 12.33% in responders vs. + 4.00% in nonresponders, *p *= 0.01).

In a linear regression model, only cardiomyopathy type and use of ACE inhibitor respectively Angiotensin II receptor blockers (ARB) had an impact on response to CRT-upgrade (Table 2). Patients with NICM presented more benefit than patients with CAD (80.4 vs. 60.3% response, *p* < 0.05). Older age as well as elevated creatinine and potassium levels were associated with the worse outcome only in univariate analysis. With regard to ECG-parameters, neither the pre-upgrade QRS-duration during RV-pacing with 177.3 ms in responders and 179.7 ms in nonresponders (*p* = 0.289) nor a reduction of QRS-duration after CRT-upgrade with 28.5 ms in responders vs. 23.1 ms in nonresponders (*p* = 0.087) had a significant impact on CRT-response. The percentage of right ventricular pacing burden before upgrade (87.6% in responders vs. 90.0% in nonresponders, *p* = 0.182) also showed no significant influence on CRT-response. Mean percentage of biventricular stimulation after CRT-upgrade was 96.84% in responders and 97.66% in nonresponders (*p *= 0.35). Duration of chronic RV-pacing before CRT-upgrade, which was known in 49.57% of patients, did also not differ bet*w*een both groups (34.48 months in responders vs. 26.18 months in nonresponders, *p* = 0.42).

**Table 2:** Results

	Responders (*n* = 79)	Nonresponders (*n* = 35)	*P* value (univariate)
Gender			0.17
Male	59 (74.68%)	29 (82.86%)	
Female	20 (25.3%)	6 (17.14%)	
Age (years)	70.82	76.23	0.014
NYHA class prior to CRT	2.91	2.99	0.102
II	6 (7.59%)	1 (2.85%)	
II–III	7 (8.86%)	3 (8.57%)	
III	62 (77.22%)	27 (77.14%)	
III–IV	4 (5.06%)	4 (11.42%)	
NYHA class post to CRT	2.16	2.99	
I	3 (3.79%)	0 (0%)	
I–II	3 (3.79%)	0 (0%)	
II	43 (54.43%)	1 (2.85%)	
II–III	26 (32.91%)	3 (8.57%)	
III	4 (5.06%)	27 (77.14%)	
III–IV	0 (0%)	4 (11.42%)	
CRT-device			0.516
Defibrillator	57 (72.15%)	23 (65.71%)	
Pacemaker	22 (27.85%)	12 (34.29%)	
Heart failure etiology			0.01
Non Ischaemic cardiomyopathy	41 (51.90%)	10 (28.57%)	
Ischaemic cardiomyopathy	38 (48.10%)	25 (71.43%)	
Ejection fraction (%) prior to CRT	34.98	33.42	0.177
EF improvement (%) after CRT^a^	12.33	4.00	0.01
QRS duration prior to CRT (ms)	177.34	179.71	0.289
QRS reduction after CRT (ms)	28.5	23.1	0.087
Right ventricular pacing burden (%)	87.56	90.89	0.182
Biventricular pacing (%)	96.84	97.66	0.350
Duration of prior RV-pacing (month)^b^	34.48	26.12	0.420
Laboratory results			
N + (mmol/l)	140.3	139.74	0.232
K + (mmol/l)	4.4	4.57	0.047
Creatinine (mg/dl)	1.42	1.71	0.033
Medication			
ß-blockers (%)	73 (92.40%)	32 (91.40%)	0.43
ACE-inhibitor (%)	72 (91.13%)	27 (77.14%)	0.021
MRA-antagonist (%)	41 (51.90%)	18 (51.43%)	0.48

^a^Available in 39 patients (26 responder, 11 nonresponder)

^b^Available in 57 patients (40 responder, 17 nonresponder)

## Discussion

The current study sought to identify characteristics associated with symptomatic response in patients exposed to chronic RVP upgraded to CRT. The primary finding in this single-center study is that only type of underlying cardiomyopathy and use of ACE-inhibitor respectively ARB had a significant impact on CRT-response in this subgroup. Other traditional factors associated with response in a de novo population receiving CRT such as female gender, QRS duration at baseline and QRS shortening after CRT did not differ significantly between responders and nonresponders. Within the investigated population with an RV-pacing burden of at least 50%, the exact degree of RVP also had no impact on symptomatic response.

Upgrade to CRT from chronic RVP represents a growing field as 23–28% of all CRT device implants were upgrade procedures [10, 11]. International guidelines recommend CRT-upgrade for patients with frequent or permanent RV-pacing, LVEF < 35% and symptomatic heart failure [1]. The Block HF trial even demonstrated the superiority of biventricular compared to right-ventricular pacing in patients with atrioventricular block and reduced LVEF < 50% [12]. Despite the above- mentioned significance, CRT-upgrade procedures were excluded from most randomized controlled CRT trials. Only the RAFT trial and the MUSTIC AF trial included RVP-paced patients and inclusion was restricted to an arbitrarily chosen QRS-width > 200 ms [13, 14].

Current outcome data for upgraded patients compared to de novo CRT implantations show conflicting results and are mainly limited due to their retrospective design. Tayal et al. [6] reported about superior survival in 50 patients with CRT upgrade compared with native LBBB patients. In contrast, Vamos et al. [7] demonstrated inferior outcomes of upgraded patients over a follow-up period of 3 years. A meta-analysis by Koztin et al. [5] showed similar all-cause mortality between de-novo and CRT-upgrade procedures.

According to this restricted outcome data, limited knowledge regarding optimal patient selection is available. As CRT-upgrade procedures are associated with a higher rate of periprocedural complications compared to de novo implantations, i.e. pneumothorax or device infection [15, 16], optimal patient selection gains even more importance.

In de novo CRT-recipients, especially NICM, QRS-duration, presence of LBBB and female gender are associated with favorable outcome [17, 18]. In contrast, our results indicate that response in patients with RVP is independent of most of these factors, except for underlying cardiomyopathy.

These findings lead to a broader candidate population for CRT upgrade compared with de novo implants. On the other hand, optimal patient selection becomes more difficult.

Presence of NICM has been shown to be one of the most robust predictors of improved outcome in de novo CRT-recipients [8]. A possible mechanism explaining poorer prognosis in ischemic heart failure patients is the presence of a large myocardial scar. Myocardial scar may lead to more complex pattern of LV activation that may not be corrected by CRT. In addition, a myocardial scar may limit LV reverse remodeling after CRT [19]. The findings of our study suggest that these considerations could be applied to chronic RVP-patients as well.

In univariate analysis, elevated creatinine and potassium levels were associated with worse outcome. These findings might be explained by a higher coincidence of renal failure and coronary artery disease. Within the observed collective patients with ICM showed a trend towards higher creatinine and potassium levels (*p* = 0.086 and 0.10).

The central significance of ACE-inhibitors in treatment of chronic heart failure has been highlighted in several randomized controlled trials [20, 21]. The results of this study underline the importance of continuing an adequate medical heart failure therapy after CRT-implantation.

In de novo patients QRS duration during intrinsic conduction, especially as typical LBBB-pattern, is another key predictor of CRT-response [22]. Prolonged QRS-duration can be considered as a correlate of electrical dyssynchrony which can be addressed by CRT. The few CRT-trials which have included RVP-patients were mostly limited to an arbitrarily chosen QRS-with cutoff of 200 ms [13, 14]. In our study mean baseline QRS-duration during RV-pacing was 177.3 ms in responders and 179.7 ms in nonresponders with no statistical significance between both groups. This is in line with the findings of Rickard et al. [23], who did not find any effect of paced QRS-width on CRT-response in 112 CRT-upgrade patients as well. In contrast, Rickard et al. showed that a reduction of QRS-width after CRT-upgrade was associated with improved outcome. This observation, however could not be confirmed in our patient collective with only a non-significant trend of QRS-reduction between both groups (28.5 ms in responders and 23.1 ms in nonresponders, *p* = 0.087).

The significance of RVP burden in CRT-patient selection remains unclear. Neither American nor European guidelines define an exact cut-off-value of RV-paced burden for considering CRT-upgrade [1, 24]. In our study, only patients with a RV-paced burden > 50% were included which is our in-house cut-off point for CRT-upgrade. Within this collective, the exact percentage of RV-paced burden had no influence on CRT-response. It remains unclear if a lower cut-off-value, e.g. 20% as proposed by other authors [25] would have influenced the results of this study.

Vamos et al. [7] hypothesized that duration of RV-pacing before upgrade may also influence CRT-response with poorer prognosis in patients with a longer history of RV-pacing and therefore more advanced cardiomyopathy. Nevertheless, there was no difference in our collective between responders and nonresponders regarding the duration of prior RV-pacing although data were only available in about half of the patients.

### Study limitations

The definition of response in our study was solely based on clinical improvement referring to the validated Heart Failure Questionnaire. Therefore, the amount of responders might have been overestimated because of the placebo effect of therapy in general. Adding solid echocardiographic parameters to the definition of response for all patients would have delivered further information on the effect of CRT. However, this study had only medium-term follow up in a medium-sized collective. Therefore, analysis of echocardiographic parameters was classified as not feasible in a prior power-analysis. Nevertheless, in cases where echocardiographic data were available, there was a significant correlation between clinical response and LVEF-improvement. The use of biomarkers such as NT-proBNP as a prognostic marker as well as an indicator for CRT-response might also put the results of study on a broader basis. However, this parameter is not routinely measured in our outpatient clinic.

The observed trend of a more pronounced QRS-reduction in responders, which is a key predictor of CRT-response in de-novo implantations might have been significant in larger patient collective.

### Conclusion

Response prediction in CRT-upgrade patients due to frequent or permanent RVP remains difficult. Only type of underlying cardiomyopathy and use of ACE-inhibitor had a significant influence on CRT-response with a favorable outcome of NICM compared to ICM. Other traditional factors in the novo CRT-population like female gender or QRS-width do not appear to apply to RVP-patients. Especially the 200 ms threshold for inclusion of chronic paced patients in past CRT-trials should be reconsidered. As long as the right ventricular pacing is above 50%, the exact pacing burden does not seem to have a significant effect on CRT-response. An exact cut-off-value for CRT-upgrade response needs still to be determined.
